# Some Clinical Aspects of Granular Kidney

**Published:** 1899-02-18

**Authors:** Samuel West


### Hospital Clinics and Medical Progress.

### SOME CLINICAL ASPECTS OF GRANULAR KIDNEY.

Abstract of the Lettsomian Lectures delivered before the Medical Society of London by Samuel West, M.D., F.R.C.P.

Granular kidney is a disease of great importance on account of its frequency, a frequency which is by no means adequately recognised. Post-mortem, it is often discovered when not suspected. It is often in itself the cause of death, even of sudden death, and it often explains why death has happened in other diseases which otherwise might not have proved fatal. During life it is often discovered unexpectedly if looked for; it is often overlooked if not suspected, and it often explains a case which has been a puzzle until granular kidney gave the key. Thus it is not only one of the most interesting of diseases, but also one of the most important.

The lesions, physical signs, and symptoms of granular kidney all alike fall into two groups—the cardio-vascular and renal. It is to be noted that while the physical signs are early the symptoms are late, and that when the latter occur the disease has already existed for some time, and the lesions are far advanced. For the diagnosis of the disease in its early stages, then, we must look to physical signs and not to symptoms.

### The Anatomical Changes.

Granular kidney is a bilateral and to a great extent a symmetrical affection, that is to say, both kidneys are similarly affected, although not necessarily to the same extent. They may be much contracted and greatly reduced in size, so that they may not weigh more than one and a half or two ounces each. But such extreme contraction as this is rare. The kidneys, it is true, are usually reduced in size and weight, but by no means to the extent that is generally believed; while in some instances, so far from being smaller than normal, they may be much above the average size and weight; in other words, there are large as well as small granular kidneys, just as there are large and small cirrhotic livers. Moreover, the kidneys are not always granular on the surface, though microscopically the interstitial change may be marked enough. The typical granular kidney, then, is small, contracted, hard, with a granular surface and often numerous cysts. On section the whole kidney is found to be cirrhotic and wasted, but the wasting affects chiefly the cortical region. The changes consist of fibroid induration and cellular degeneration. As to the terms "white" and "red" granular kidney, as expressive of different kinds of disease, and the use of phrases denoting different origins, the one form of kidney being called the *contracted white* and the other the *contracted red*, although the two forms certainly exist there seems to be no proof that they have different origins, or even that the small white is but the later stage of the large white kidney.

It is, therefore, much better to speak of them as the small white and the small red than to use terms which suggest different origins, especially as clinically no distinction at all can be drawn between them; that is to say, that with exactly the same clinical symptoms we may find the kidneys in this one case white and in the other case red.

### Interstitial Nephritis which is not Granular Kidney.

Granular kidney is often described as chronic interstitial nephritis. But the converse is not true; all forms of interstitial nephritis are not necessarily granular kidney—as, for example, the unilateral form which results from obstruction to the ureters; patchy fibrosis resulting from infarcts or gummata; and also those degenerate kidneys which are met with in connection with advanced atheromatous disease or with the chronic gout of elderly persons, for these, although markedly interstitial, need not necessarily be granular and need not run the clinical course of granular kidney.

### Relations with Inflamed Kidney and with Degenerate Vessels.

In most recent writings granular kidney is divided into two forms, the arterio-sclerotic and the renal. This is very inconvenient and confusing, and suggests a marked difference between the two forms which, clinically, does not exist. It has, however, this advantage, that it fixes attention upon the question which is still at issue—namely, whether it is in the vessel or in the kidney itself that the primary causes of the disease are to be sought. Acute nephritis attacks the cells primarily and chiefly, but the interstitial tissues are always involved to some extent, and the more so the longer the disease has existed. In course of time a small-celled infiltration is found, in parts at any rate, following the lymphatics and surrounding the blood-vessels, and this small-celled infiltration may in time end in the production of connective tissue. Admitting, then, that pathological connective tissue contracts in the kidney or elsewhere, we should expect the large white kidney (the later form of the large red kidney) to undergo a certain degree of contraction, and of this we have frequent pathological proof. Theoretically it is even conceivable that the contraction might go much further, and that the large white kidney might pass into the small white, and might even end in the small red kidney. But if this actually occurred frequently, it ought to be capable of easy clinical proof, for the symptoms of acute nephritis are not such as are likely to be overlooked. Such cases, however, although on record, are certainly rare, and many of them, on investigation, are not so conclusive as they at first sight appear.

Again, tracing back the history of cases of granular kidney, it is quite unusual to obtain any history of symptoms which would in any way justify the diagnosis of acute nephritis, and even where such a history is obtained it is no proof that prior to the acute nephritis the kidney was sound. Indeed, there is some reason to believe that not infrequently when acute nephritis occurs in the adult, signs of a pre-existing granular kidney would be found if they were looked for. It is evident, then, that but few cases of granular kidney can be traced either back to or forward from an initial acute nephritis. It follows, therefore, that most cases of granular kidney must be referred to some other origin.

If, then, we turn to the arteries we find that changes in them are widespread, in fact, universal, throughout the whole body in cases of granular kidney. As to the nature of these vascular changes dispute has long raged, one side maintaining that it was of the nature of hypertrophy, the other of a degeneration. The facts are that both changes are found, the hypertrophic changes in the earlier stages and the degenerative in the latter. So far as the hypertrophy of the arteries is concerned it is probably, like that of the heart, secondary—both heart and arteries hypertrophying together for the purpose of assisting the circulation to overcome some obstruction in the small peripheral arteries or capillaries. If, however, the changes in the vessels be primary we ought to have evidence of vascular disease before we get evidence of kidney disease, and there ought to be such cases in which at the time of death the kidneys are found not in a condition of granular change. But such cases, although described, are very rare, and thus from whatever point of view we regard granular kidney we come to the same conclusion, namely, that it is a disease *sui generis*.

### Symptoms.

Granular kidney is a very insidious disease, and for a long while presents no symptoms at all, and can be only recognised by physical signs. When, however, symptoms do show themselves they fall, like the lesion, into two groups, the cardio-vascular and the renal. The cardiac symptoms are those of heart failure more or less pronounced; the vascular symptoms are the more or less mechanical efforts of the vascular lesion, the renal symptoms, which are the latest to develop, and consist chiefly of hæmorrhage and its results; while may be described as acute and chronic renal toxæmia, or, perhaps, still better as uræmia and chronic renal cachexia. Granular kidney is often said to be a disease of middle life, and this is true in that the symptoms usually become evident then; but the disease must have commenced long before, and in some cases we know that it may be fatal even in quite early years. The physical signs in the early stages are high tension and thickening of the arteries, hypertrophy of the heart, and albuminuria.

### Hypertrophy of the Heart.

Post-mortem evidence would point to hypertrophy of the heart as a most important sign of granular kidney, but it is not as useful a sign as might therefore be supposed, partly because in early stages it is not easily recognised, and partly because it may be produced by other causes as by atheroma of the vessels. As for accentuation of the second sound of the heart, this is a sign of small value, for while it occurs whenever the arterial tension is raised, from whatever cause, it will be absent even in granular kidney when the tension fails.

### Thickened Arteries.

Thickening of the arteries is one of the cardinal signs of the disease, and is never absent in advanced cases. Now, thickening of arteries is not uncommonly found even in young people without other symptoms, and the probabilities are in favour of persons who are in this condition being really in the early stage of granular kidney.

### High Arterial Tension.

In well marked granular kidney the high-tension pulse is well recognised. The arteries feel abnormally full as well as abnormally tense between the beats. When the heart begins to fail the artery may be less distended, and therefore the wave not so high, but its round top will persist, and when in the end the tension falls the thickening of the arteries can still be made out. But it must be remembered that though a high tension under ordinary circumstances is of itself mischievous, it is not so with granular kidney. The patient is best without granular kidneys, but if the kidneys be granular it is better that the tension should be high than low. What, then, is the diagnostic value of high tension in a person in whom there is not yet other evidence by which to diagnose granular kidney? Recent study of the retinal changes associated on the one hand with these cases of high tension and thickened artery and on the other with granular kidney tend strongly to confirm the conclusion arrived at by clinical observation to the effect that these cases of high tension and thickened arteries in the young are indeed the initial stages of granular kidney.

